# Female rats adopt a safety-first strategy in a high-conflict platform mediated avoidance task

**DOI:** 10.3389/fnbeh.2026.1758605

**Published:** 2026-02-18

**Authors:** Adriano E. Reimer, Christina J. Li, Steven M. Hu, Delilah Pineda, Jason L. Chang, Michael R. Angstman, Evan M. Dastin-van Rijn, Alik S. Widge

**Affiliations:** Department of Psychiatry and Behavioral Sciences, University of Minnesota, Minneapolis, MN, United States

**Keywords:** active avoidance, anxiety disorders, approach-avoidance conflict, estrous cycle, platform mediated avoidance, sex differences

## Abstract

Maladaptive avoidance is a central feature of many mental disorders, particularly stress- and anxiety-related disorders. Those disorders are more prevalent in women, suggesting that there may be sex differences in avoidance propensity. Sex differences have been documented in threat conditioning, but not in active avoidance paradigms, despite their potential clinical relevance. Preclinical research has historically focused on males, limiting our understanding of the neurobiological mechanisms underlying sex differences in threat responses. To address this gap, we investigated sex-specific strategies in adult Long Evans rats (10 female, 9 male) using a platform-mediated avoidance (PMA) task that created a high-conflict choice between reward-seeking and safety. Behavior was tracked over 25 days, with analyses focusing on a stable performance phase (days 20–25) objectively defined using change point analysis. Females consistently prioritized safety, spending significantly more time foregoing reward to avoid foot shock and retreating earlier to the safe zone. Males engaged in more persistent reward-seeking despite the risk of shock. This difference was not driven by differential reward motivation. Furthermore, female strategies were not significantly modulated by the estrous cycle. These results were consistent in a pre-registered replication study. Thus, male and female rats employ fundamentally different strategies to resolve approach-avoidance conflict: females adopt a robust, safety-first strategy, while males demonstrate a risk-prone, reward-oriented approach. Identifying the neural mechanisms underlying these differences may guide more targeted interventions for anxiety and trauma-related disorders.

## Introduction

Women are substantially more likely than men to be diagnosed with stress- and anxiety-related disorders, including post-traumatic stress disorder and generalized anxiety disorder (Kessler et al., 2005; McLean et al., 2011; Haering et al., 2024). These conditions are highly comorbid, share overlapping symptoms (Price and van Stolk-Cooke, 2015; Afzali et al., 2017), and rank among the most prevalent mental health challenges globally, contributing to significant societal and economic costs (Fineberg et al., 2013; Trautmann et al., 2016). Despite the clear sex disparity in prevalence, the underlying neurobiological mechanisms driving these differences remain poorly understood (Lebron-Milad and Milad, 2012; Day and Stevenson, 2020).

A core behavioral symptom and transdiagnostic feature across these disorders is maladaptive avoidance, where individuals excessively avoid situations or stimuli perceived as threatening (Dymond, 2019). Avoidance frequently arises from attempts to regulate intense negative emotions, including preventing contact with sources of distress by avoidance of external triggers and the avoidance of internal thoughts and feelings (Barlow, 2008; Ellard et al., 2010). Because avoidance can severely impair daily functioning, it is a key target for clinical intervention (Salters-Pedneault et al., 2004; Blakey and Abramowitz, 2016). Examining the basis of sex differences in the expression of avoidance, which allows for better understanding of the dysfunctional mechanisms underlying the mental disorders, is therefore a critical step toward developing more effective, personalized treatments.

Rodent models have been instrumental in elucidating the neural circuits of threat conditioning and avoidance (LeDoux, 2000; Maren, 2001; Bienvenu et al., 2021; Diehl et al., 2024), yet preclinical research has historically focused almost exclusively on male subjects (Beery and Zucker, 2011; Dalla and Shors, 2009; Shansky, 2018). This persistent exclusion of females has limited our ability to model the full spectrum of anxiety pathology and has created significant gaps in our knowledge of the female neurobehavioral response to threat (Shansky, 2019; Shansky and Murphy, 2021).

The limited studies that have included both sexes reveal important sexual dimorphisms. For instance, female mice can demonstrate more persistent avoidance following extinction training (Halcomb et al., 2024), and both female rats and mice often employ different defensive response strategies compared to males, such as active darting or “anxioescapic” behaviors instead of passive freezing (Gruene et al., 2015a; Shanazz et al., 2022; Halcomb et al., 2024). This divergence may reflect fundamentally different cognitive strategies for assessing risk. For example, in value-based decision-making, females are more likely to use a steady, long-term “less risky” strategy, whereas males often change their approach based on recent outcomes (Chen et al., 2021a, 2021b). Such findings suggest that sex-based variations could arise from sex-biased cognitive approaches (Grissom et al., 2024). Sex-biased behaviors are often assumed to arise from gonadal hormones, but hormonal contribution to avoidance-related phenomenas is not straightforward. For example, although hormonal fluctuations across the estrous cycle in females can modulate the consolidation of safety learning (Maeng et al., 2017) and increase vulnerability to deficits in contextual threat extinction in a pharmacological OCD model (Reimer et al., 2018), they show little effect on other behaviors. These include, for example, open-field exploration (Levy et al., 2023), and in interactions in the resident-intruder assay (Zeng et al., 2023).

A second challenge in existing literature is that it depends heavily on paradigms that may not model clinically relevant avoidance (Aupperle and Paulus, 2010; Kirlic et al., 2017; Ball and Gunaydin, 2022). Avoidance tasks are typically categorized as passive (withholding an action to avoid threat) or active (performing one). Focusing on the latter, many standard active avoidance paradigms, like the shuttle box, have translational limitations (Diehl et al., 2019). The shuttle avoidance (Mowrer and Lamoreaux, 1946; Krypotos et al., 2015), for example, requires an animal to repeatedly re-enter a compartment where it was just shocked. This creates a condition where there is no well-defined safe location, leading to an ambiguous response: it promotes freezing behavior that directly competes with, and can be mistaken for, the avoidance strategy being measured. Furthermore, these tasks typically omit any cost for the avoidant action, failing to model the clinically relevant situations where individuals sacrifice valuable opportunities by engaging in excessive, maladaptive avoidance that severely impairs their daily functioning (Aupperle et al., 2015; Pittig et al., 2021).

More recently, the Platform Mediated Avoidance (PMA; Bravo-Rivera et al., 2014) task was developed to address these issues, allowing for a less ambiguous behavioral response by providing a distinct safe zone while also incorporating a cost–benefit component. In PMA, animals may food-seek by pressing a lever, but to avoid a signaled foot shock, must retreat to an insulating platform far from the lever. However, this paradigm models a specific type of conflict where the threat and reward are separated in time; an animal can simply wait for the threat to pass before safely resuming reward-seeking. While this is a valuable model, it does not fully capture the decision-making characteristic of clinical anxiety, where valuable opportunities are contingent upon enduring a perceived threat. A more direct model requires forcing an explicit trade-off where the reward is only available in the presence of the threat. Investigating the potentially dimorphic strategies that emerge under such intense conditions - which can reflect differences in threat-response regulation, decision-making, or both (Orsini et al., 2016; Greiner et al., 2019; Xu et al., 2022) - is a crucial step toward understanding the neurobiological basis of avoidance circuits and how they may be altered in clinical populations (Olff, 2017; Panayiotou et al., 2017).

Therefore, the present study employed a high conflict PMA task similar to Bravo-Rivera et al. (2021), designed to create the type of intense approach-avoidance conflict needed to reveal these strategies. In our paradigm, animals could only access rewards when a threat was imminent, forcing a choice between reward-seeking and safety. Aside from having more face resemblance to clinical avoidance, this increased conflict may invoke more deliberative, value-based decision-making, which may be revealed in vicarious behaviors (Papale et al., 2012; Schmidt et al., 2013). We tracked the development of avoidance and approach behaviors over 25 days to examine how strategies were acquired and expressed once they stabilized, allowing us to compare both the learning dynamics and end-stage behavioral patterns between sexes.

Based on the clinical prevalence of anxiety disorders in women and preclinical evidence of dimorphic threat responses, we hypothesized that male and female rats would adopt distinct strategies. Specifically, we predicted that females would exhibit greater avoidance behavior, prioritizing safety at the cost of reward, whereas males would demonstrate more risk-insensitive reward-seeking. We also conducted exploratory analyses to determine whether behavioral strategies in females were modulated by the estrous cycle, given the putative influence of reproductive hormones on threat-detection / threat-response in rodents (Reimer et al., 2018; Maeng et al., 2017).

## Methods

### Preregistration

The first cohort (4 female, 4 male) was not preregistered. To test whether the exploratory findings from that cohort were replicable, the experimental design and analysis plan for the second cohort (6 female, 5 male) were preregistered on the Open Science Framework (https://osf.io/gav3h) prior to data collection. The sample size was determined via power analysis for repeated measures ANOVA assuming a small effect size (f^2^ = 0.1), alpha of 0.05, and power of 0.80 (full details in preregistration), used as a conservative approximation for the planned GLMMs.

### Animals

All experiments were conducted in accordance with the NIH Guidelines for the Care and Use of Laboratory Animals and were approved by the University of Minnesota Institutional Animal Care and Use Committee (IACUC, protocol 2,303-40918A). A total of 19 adult Long Evans rats (10 female, ~250 g; 9 male, ~350 g; ~3 months old), purchased from Charles River Laboratories (Wilmington, MA), were used in this study. Upon arrival, rats were pair-housed under a 12-h reverse light/dark cycle (lights off at 9:00 a.m.) and allowed to acclimate for at least 7 days. Following acclimation, they were individually housed and handled daily for 7 consecutive days. Individual housing was maintained throughout the experiment to ensure methodological consistency with prior studies characterizing PMA (Bravo-Rivera et al., 2014; Bravo-Rivera et al., 2021). To maintain motivation for the sucrose reward, rats were food restricted to 90% of their baseline body weight. Their weight was monitored daily, and food allotment was adjusted to maintain this target weight (not falling below 85% of baseline). All behavioral testing occurred during the dark phase under red light.

Animals were tested in two cohorts. The first cohort (4F, 4 M) was run prior to preregistration. The second, preregistered cohort (6F, 5 M) served as a replication. Behavioral modeling was conducted separately for each cohort to preserve the confirmatory nature of the preregistered analyses. Data from both cohorts were combined for estrous-stage analyses, which were designated as exploratory in the preregistration, to increase statistical power.

### Apparatus

Behavioral testing was conducted in modified operant chambers (40.6 cm × 25.4 cm × 44.0 cm; Lafayette Instruments, Lafayette, IN) housed within sound-attenuating cubicles. Each chamber featured a grid floor capable of delivering scrambled footshocks, a retractable lever with a cue light and speaker, and pellet dispenser and trough on one wall, and an acrylic safety platform (10.2 cm × 25.4 cm) on the opposite wall. Sessions were recorded by digital video cameras (DMK 37AUX287, The Imaging Source, NC) mounted above the behavioral chamber. A computer and interface unit (Lafayette Instruments) was connected to the chambers, and all task parameters, stimulus presentation, and data acquisition were controlled by in-house developed behavior control software Pybehave (Rijn et al., 2024).

### Procedures

#### Platform mediated avoidance task

**Figure 1:**
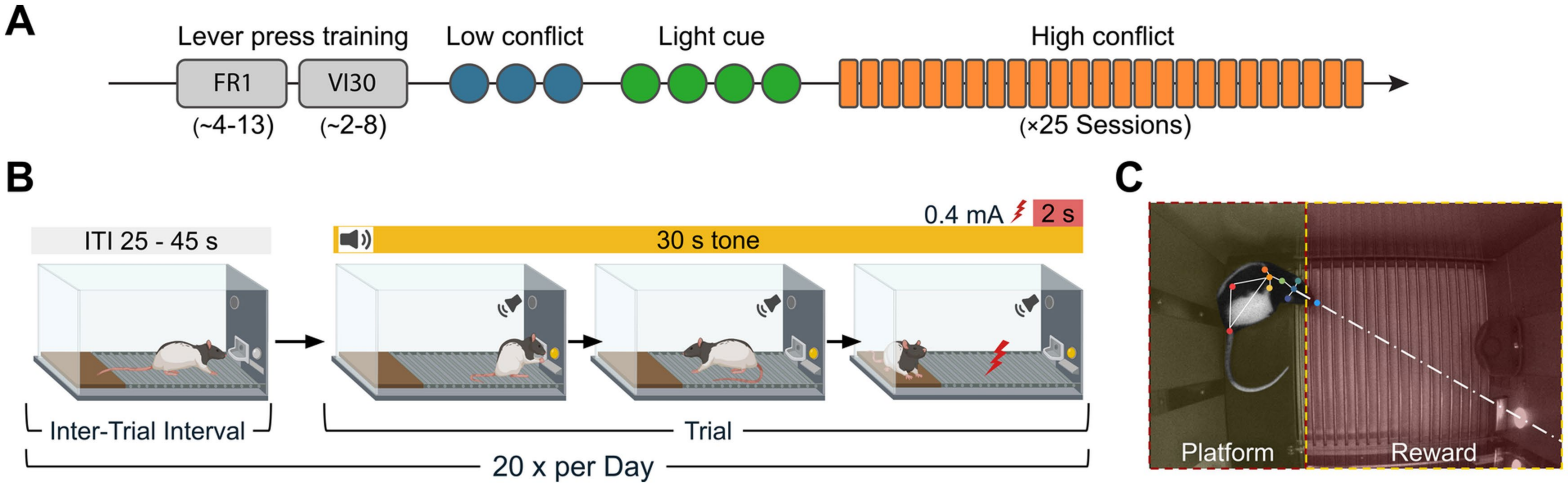
Experimental timeline and platform mediated avoidance (PMA) task paradigm. **(A)** Experimental timeline: after habituation and once target weight was reached through food restriction, rats were trained to lever press for a food reward on fixed-ratio (FR1: 4–13 sessions) and variable-interval (VI30: 2–8 sessions) schedules. This was followed by low conflict (3 days), light cue association (4 days), and high conflict (25 days) phases. **(B)** High conflict PMA task: each daily session began with a 30-s pre-trial interval and consisted of 20 trials (variable inter-trial interval, ITI, 25–45 s), concluding with a 45-s post-trial interval. A trial begins with the onset of a 30-s auditory tone. Only during this tone, the rat can press the lever to receive a food reward but must retreat to the safe platform to avoid a footshock (0.4 mA for 2 s) delivered through the grid floor at the end of the tone. **(C)** Still frame showing tracked body points using DeepLabCut. Zones of interest (platform and reward, shaded in yellow and red, respectively). The frame also illustrates the assessment of reward attentiveness, where the animal’s head is oriented toward the reward zone (indicated by the eyeline vector).

The behavioral protocol consisted of three sequential stages: (1) initial lever press training, (2) conditioning of separate threat and reward cues, and (3) an extended high-conflict PMA task (Figure 1A).

First, all animals underwent operant conditioning to press a lever for 45-mg sucrose pellets in 40-min daily sessions. Training began on a fixed-ratio 1 (FR1) schedule and advanced to a variable-interval 30-s (VI30) schedule after an animal achieved a rate of at least 10 presses within the first 10 min of a session. The same criterion was required on the VI30s schedule before proceeding. The number of sessions required to reach these criteria varied by animal (FR1: 4–13 sessions; VI30: 2–8 sessions).

After lever-press acquisition, rats underwent two distinct pre-training phases to establish key associations. First, in a three-day, low-conflict conditioning phase, a 6 kHz warning tone was paired with a co-terminating 2-s, 0.4 mA footshock over twenty 30s trials (Inter-Trial-Interval, ITI: 25–45 s). The lever remained continuously available during the ITI. Next, animals were subjected to a four-day reward-cue phase, in which the lever and an associated cue light were made accessible only during the 30 s trial periods, with no tones or shocks delivered. This trained the animals to associate the light-cued trial period with reward availability.

The final phase was a 25-day high-conflict PMA task. Each session began with a 30-s pre-trial interval and concluded with a 45-s post-trial interval following the last tone/shock presentation, after which animals were removed. The trial structure (twenty 30s trials; 25–45 s ITI) was maintained. The food lever and its light cue were now accessible only during the presentation of the tone, which still co-terminated with the footshock. This design created a direct approach-avoidance conflict, forcing animals to choose between approaching the lever for a reward and retreating to the platform for safety (Figure 1B). At the end of each trial, the lever was retracted, becoming unavailable during the ITI. While consistent with the paradigm used by Bravo-Rivera et al. (2021), our protocol adds lever retraction to prevent reward-seeking during the safety of the ITI.

#### Estrous cycle staging

Following each behavioral session, vaginal lavage was conducted on female rats to determine estrous stage. It was performed using a pipette containing approximately 100 μL of sterile saline. Saline was gently flushed in and out of the vaginal canal several times to collect epithelial cells. The sample was then transferred to a glass slide. The slides were left to dry in room temperature followed by staining (DipQuick Stain Fixative, Stain Solution, and Counter Stain, JorVet, CO) and examination under a light microscope for cytological assessment (Cora et al., 2015). To control for stress associated with this procedure, male rats were held in the same restraint position for a similar duration after each session.

### Behavioral and statistical analysis

#### Pose estimation

Animal body position was tracked using DeepLabCut (v3.0RC10; Mathis et al., 2018, Nath et al., 2019). A ResNet-50-based neural network (Insafutdinov et al., 2016; He et al., 2016) with default parameters was used on 1,230 manually labeled frames from 108 video fragments including different animals/sessions (95% training, 5% testing), for 6 shuffles. The final trained network achieved a test error of 2.61 pixels and a training error of 1.6 pixels (image size: 720 × 540 pixels) with a p-cutoff of 0.6.

#### Post-processing

All subsequent behavioral data processing was performed in the RStudio environment (v2025.5.1.513; Posit team, 2025) using R (v4.5.1; R Core Team, 2025). Data wrangling and transformation were conducted primarily using the tidyverse (Wickham et al., 2019; v2.0.0), including its component packages dplyr (Wickham et al., 2019; v1.1.4), tidyr (Wickham et al., 2024b; v1.3.1), tibble (Müller and Wickham, 2025; v3.3.0), stringr (Wickham, 2023; v1.5.1), purrr (Wickham and Henry, 2025; v1.1.0), readr (Wickham et al., 2024a; v2.1.5), and glue (Hester and Bryan, 2024; v1.8.0). In addition, data.table (Barrett et al., 2025; v1.17.8) was used for data aggregation.

The behavioral chamber was divided into two zones: platform and reward (Figure 1C). Events (tone, shock, lever press) were extracted from PyBehave log files. An animal’s location was determined by the coordinates of its lower back, defining the time spent in each zone. In addition to physical location, reward attentiveness was calculated as the total time an animal spent either within the reward zone or oriented toward it from outside the zone, a metric designed to quantify the deliberative “stretch-attend” postures characteristic of Vicarious Trial and Error during risk-assessment (Redish, 2016). Head orientation was determined by calculating a forward-projecting vector from the animal’s head-center through its nose on a by-frame basis. If this vector intersected with the reward zone’s coordinates, the frame was counted as “looking at the reward.”

#### Change point analysis

We expected that sex differences might best be revealed once animals had reached stable task behavior, rather than during periods where they were still identifying preferred strategies. To objectively identify the onset of that stable, end-stage behavior, Bayesian change point analysis was applied to the daily averages of three key metrics from Cohort 1: percent time on the platform, reward attentiveness, and total bar presses. Using the mcp package in R (Lindeløv, 2020; v0.3.4), performance for each metric and sex was modeled with a three-segment trajectory: 1) a flat baseline, 2) a quadratic learning phase, and 3) a final flat stable phase (model: DailyAvg ~ 1, ~ 0 + DAY + I(DAY^2), ~ 1). This extended learning phase is characteristic of the PMA paradigm, which requires a prolonged period for the initial freezing response to subside and for competing behaviors, such as reward-seeking, to stabilize (Bravo-Rivera et al., 2014; Martínez-Rivera et al., 2020). The second change point, marking the transition into the stable phase, was estimated for each model. The latest of these change points across all metrics and both sexes was Day 19 (see Results). Therefore, performance on days 20–25 was defined as the stable phase for both cohorts.

#### Behavioral modeling

Stable-phase behavior was analyzed using generalized linear mixed models (GLMMs) in R, with models fit separately for each cohort (Bolker et al., 2009). The high-conflict design of the task forcing a choice between the safety of the platform and the reward at the lever, produced bimodal data distributions. Specifically, many trials resulted in mutually exclusive behavioral outcomes, where an animal either remained on the platform for the entire duration (resulting in 100% platform time and zero bar presses) or committed to reward-seeking by approaching the lever. To properly capture this structure, we selected two-part models that separate the binary choice to engage in a behavior from the subsequent frequency or duration of that behavior (Fisher et al., 2017; Zhu et al., 2017). Based on data characteristics from Cohort 1, four primary dependent variables were modeled

1. Bar Presses: Modeled using a zero-inflated negative binomial (ZINB) GLMM. The model’s zero-inflation component estimated the probability of a rat not approaching the lever at all within a trial, while the negative binomial component modeled the number of bar presses on trials where it did approach.
2. Proportion of Time on Platform: Modeled using a hurdle-beta GLMM. A binomial model first predicted the probability of spending any time off the platform (hurdle), followed by a beta regression on the proportion of time for trials where the animal did not spend 100% of the time on the platform.
3. Reward Attentiveness: Modeled using a beta GLMM to analyze the proportion of trial time the animal was oriented toward the reward zone.
4. Latency to the platform: To assess the timing of the avoidance strategy, we modeled the latency to enter the safe platform zone using a Gamma GLMM with a log link function.

For both proportion-based measures (platform time and attentiveness), values were transformed using the Smithson and Verkuilen (2006) method to fit the open (0,1) interval required for beta regression.

Each model included fixed effects for sex (female = 0, male = 1), day (continuous), trial (1-20, continuous), handler (categorical), previous shock history on that day (SH; binary). We included handler as a variable because avoidance behaviors can be highly sensitive to a rat’s overall level of comfort, which could easily be influenced by small changes such as handlers’ vocal tone or choice of perfume. The same three handlers performed all procedures in both cohorts, following a standardized protocol for animal transport, weighing, and lavage/handling. Handlers were assigned based on daily availability, resulting in a quasi-randomized rotation that ensured each handler tested every animal, preventing confounding of handler identity with sex or individual animal phenotype. To account for repeated measures, animal ID was included as a random intercept. An important consideration for the analysis was the inherent correlation between sex and body weight, as adult male and female rats have distinct weight distributions. To create a predictor for relative size that was independent of sex, we transformed each animal’s absolute weight by z-scoring it against the mean and standard deviation of all weights from its respective sex, calculated across the stable phase (days 20-25). This average z-scored weight was then included as a fixed effect in the models. The preregistered analysis plan also included an interaction term for sex-by-weight. However, post-hoc multicollinearity diagnostics revealed that the sex-by-weight interaction introduced statistical instability, indicated by the Variance Inflation Factors exceeding 5 (Kim, 2019). Therefore, in a deviation from the preregistration, this term was removed from the final reported models to ensure their validity and the reliability of the coefficients

Baseline lever-pressing rates from the combined cohorts during the Light Cue phase were analyzed using a negative binomial GLMM. This model included fixed effects for sex and session, with animal ID as a random intercept.

All continuous predictors were normalized to a 0-1 scale. Statistical significance was set at *p* < 0.05. Models were fit using the glmmTMB package (Brooks et al., 2017; v1.1.11) and lme4 package (Bates et al., 2015; v1.1.37), with alternative optimization algorithms accessed via optimx (Nash and Varadhan, 2011; v2025-4.9) when convergence issues arose. Residual and model diagnostics were performed using DHARMa (Hartig et al., 2024; v0.4.7), performance (Lüdecke et al., 2021a; v0.15.0), and see (Lüdecke et al., 2021b; v0.11.0). Robust variance estimates were checked using sandwich (Zeileis et al., 2020; v3.1.1) and lmtest (Zeileis and Hothorn, 2002; v0.9.40).

#### Estrous based analysis

To explore hormonal influences on stable behavior, we conducted an exploratory analysis using trial-level data from female rats (n = 10) across both cohorts during the stable phase (Days 20-25). Given the continuous daily testing design, where sessions were conducted regardless of estrous phase, the distribution of the stages was unbalanced, reflecting the natural duration differences across cycle phases. Daily cytological classifications were therefore grouped into a binary “Hormone_Level” factor: “High-Hormone” (proestrus, estrus) and “Low-Hormone” (metestrus, diestrus), following Reimer et al. (2018), with the latter as reference category. Across the stable phase (Days 20-25), this yielded 56 sessions from 10 female subjects: 23 classified as High-Hormone (contributed by all 10 subjects) and 33 classified as Low-Hormone (contributed by 9 subjects). We applied the same GLMM framework used in the primary analysis, with Hormone_Level replacing Sex as the primary predictor of interest. The Reward Attentiveness (beta GLMM) and Platform Time (hurdle-beta GLMM) models successfully converged with all preregistered covariates. However, the Bar Presses (ZINB) model initially failed to converge due to statistical instability from the reduced female-only sample size. To achieve a stable model while preserving the most influential variables, we removed the average z-scored weight term from the conditional component, as this was the least consistent predictor across cohorts in the main analysis. The zero-inflation component retained all covariates. All models included animal ID as a random intercept.

#### Visualization and reporting

Figures were generated using ggplot2 (Wickham, 2016; v3.5.2), with palettes from RColorBrewer (Neuwirth, 2022; v1.1.3) and additional layers from ggnewscale (Campitelli, 2025; v0.5.2) and ggsignif (Ahlmann-Eltze and Patil, 2021; v0.6.4). Plot layouts were composed with patchwork (Pedersen, 2025; v1.3.1). Tables were formatted with gt (Iannone et al., 2025; v1.0.0). Model outputs were tidied for reporting using broom.mixed (Bolker and Robinson, 2024; v0.2.9.6).

## Results

### Change point analysis

**Figure 2:**
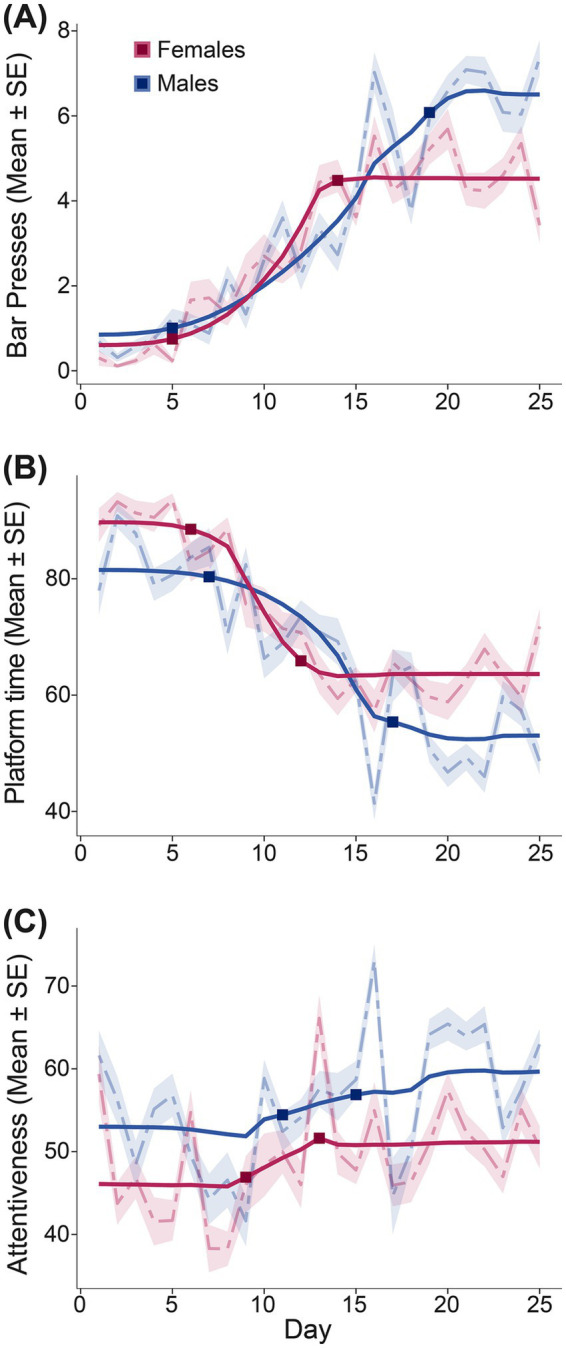
Determination of stable behavior using change point analysis. The onset of stable performance in Cohort 1 (*n* = 8; 4 females, 4 males) was modeled across three key behaviors: **(A)** Total daily bar presses, **(B)** Percent of time spent on the platform, and **(C)** Percent of time attentive to the reward zone. Dotted lines represent observed means ± standard error; solid lines show the mean posterior prediction of the fitted segmented regression model. Square markers denote estimated change points.

To objectively define the transition from learning to stable behavior, we conducted a Bayesian change point analysis on the daily performance metrics of the initial cohort (Cohort 1). We fit a three-segment piecewise regression model (flat baseline, quadratic learning, flat stable phase) to three behaviors: avoidance, reward attentiveness, and bar presses. Models were run for 700,000 iterations across 7 chains to ensure convergence. For females, the transition to the stable plateau occurred at Day 11.36 for avoidance (95% CI: 8.00–14.96), Day 12.46 for reward attentiveness (95% CI: 6.20–25.00), and Day 13.07 for bar presses (95% CI: 9.44–17.00). Males exhibited a more extended learning phase, with behavior stabilizing at Day 16.70 for avoidance (95% CI: 13.00–22.57), Day 14.74 for reward attentiveness (95% CI: 8.00–24.03), and Day 18.92 for bar presses (95% CI: 14.71–23.00). To establish a conservative window ensuring that all animals had reached a performance asymptote, we defined the stable phase based on the latest change point. With the final transition occurring at Day 19 for male bar pressing, all subsequent analyses were conducted on data from experimental days 20–25 (Figure 2).

To ensure that sex differences in the PMA task were not driven by baseline differences in reward motivation or motor capability, we analyzed lever-pressing behavior during the Light Cue phase, where the lever was available during trials but no footshocks were delivered. A GLMM on the combined cohorts revealed no significant effect of sex on the number of bar presses (*β* = 0.20, *z* = 1.40, *p* = 0.161), indicating that males and females exhibit similar rates of responding before the high conflict phase.

### Initial study and replication

To investigate sex differences in avoidance and reward-seeking strategies during the stable phase of behavior (days 20–25), we analyzed three key metrics: bar presses, avoidance (proportion of time on the platform), and reward attentiveness. Additionally, to quantify the timing of the avoidance strategy, we included a fourth metric: Latency to the platform. GLMMs were fit separately for Cohort 1 and Cohort 2.

#### Cohort 1

##### Reward-seeking behavior (Bar presses)

**Figure 3:**
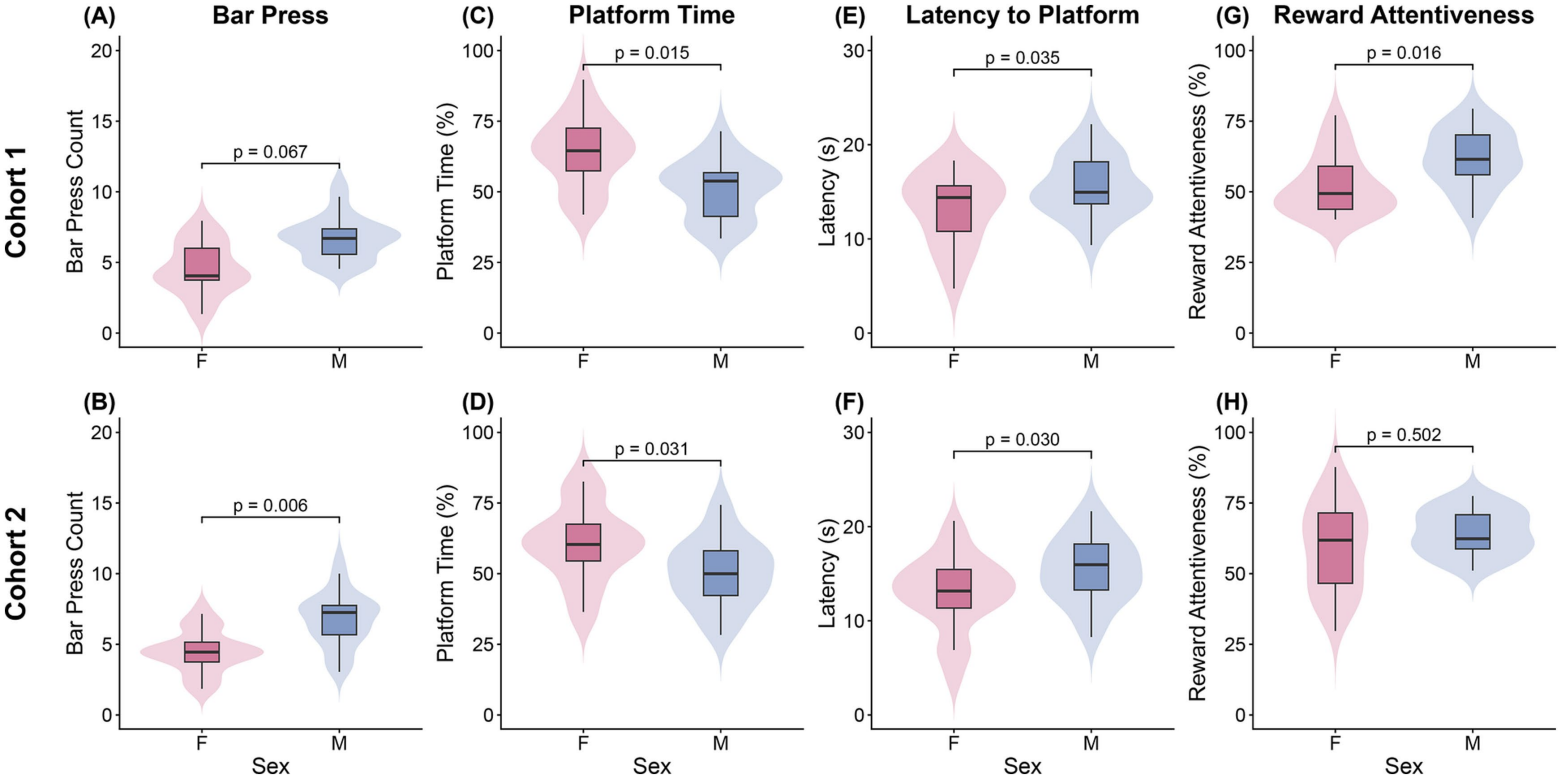
Sex differences in stable-phase performance in the high conflict task. Performance during days 20–25 is shown for Cohort 1 (top row; *n* = 4F, 4 M) and the replication Cohort 2 (bottom row; *n* = 6F, 5 M). Violin plots with overlaid boxplots comparing females and males across three metrics: **(A,B)** Bar press count, **(C,D)** Platform time (%), **(E,F)** Latency to platform (s), and **(G,H)** Reward attentiveness (%). *p*-values from statistical comparisons between sexes are displayed above groups.

Analysis with the ZINB model showed a non-significant increase in male reward seeking compared to females (*β* = 0.15, *z* = 1.83, *p* = 0.067; Figure 3A; Supplementary Table 1). Shock History (SH, any shock that day) was a significant negative predictor of bar pressing (*β* = −0.33, *z* = −7.97, *p* < 0.001). Additionally, the animal’s average z-scored weight was a significant positive predictor of bar presses (*β* = 0.26, *z* = 2.83, *p* = 0.005). The zero-inflation portion of the model, which predicts the likelihood of an animal making no presses, showed that both SH (*β* = 0.66, *z* = 2.84, *p* = 0.005; Supplementary Table 2) and day (*β* = 0.93, *z* = 2.60, *p* = 0.009) significantly increased the probability of complete inaction in a trial.

##### Avoidance behavior (platform time)

Avoidance behavior was analyzed with a two-part hurdle-beta model. The first part (binomial hurdle) revealed no sex difference in the decision to leave the platform at any point during a trial (*p* = 0.124; Supplementary Table 3). However, the second part of the model (beta), analyzing the trials where animals actually left the platform, revealed a significant main effect of sex, with males spending less time on the platform than females (*β* = −0.30, *z* = −2.44, *p* = 0.015; Figure 3C; Supplementary Table 4). Avoidance in this cohort was also significantly increased by SH (*β* = 0.68, *z* = 9.43, *p* < 0.001) and day (*β* = 0.27, *z* = 2.11, *p* = 0.035), while being significantly decreased by being in a later trial (*β* = −0.33, *z* = −3.22, *p* = 0.001) or having a heavier weight (*β* = −0.54, *z* = −4.33, *p* < 0.001).

##### Latency to safety

The Gamma GLMM revealed a significant main effect of sex, with males exhibiting longer latencies to reach the platform compared to females (*β* = 0.16, *z* = 2.11, *p* = 0.035; Figure 3E; Supplementary Table 5). This effect persisted after controlling for body weight (*β* = 0.32, *z* = 3.72, *p* < 0.001), which was a significant positive predictor. Latency was also significantly reduced by SH (*β* = −0.38, *z* = −9.74, *p* < 0.001) and day (*β* = −0.18, *z* = −2.64, *p* = 0.008). Conversely, latency increased later in the session (Trial: *β* = 0.25, *z* = 4.26, *p* < 0.001).

##### Reward attentiveness

The beta GLMM for reward attentiveness revealed a significant main effect of sex. Males were significantly more attentive to the reward zone than females (*β* = 0.42, *z* = 2.40, *p* = 0.016; Figure 3G, Supplementary Table 6). Reward attentiveness was also significantly influenced by the handler (H2 vs. H1: *β* = 0.17, *z* = 2.16, *p* = 0.031) and reduced by SH (*β* = −0.27, *z* = −4.56, *p* < 0.001).

We then pre-registered these findings as predictions for a replication analysis, powered to detect the effect sizes observed in Cohort 1.

#### Cohort 2

##### Reward-seeking behavior

The ZINB model revealed a significant main effect of sex, with males exhibiting a higher rate of bar pressing than females (*β* = 0.44, *z* = 2.74, *p* = 0.006; Figure 3B, Supplementary Table 1), i.e., directionally the same as Cohort 1 but achieving significance in this larger sample. Similar to the first cohort, SH significantly reduced bar pressing (*β* = −0.15, *z* = −4.59, *p* < 0.001). Additionally, bar pressing was significantly higher for both handling groups H2 and H3 compared to the reference (H2 vs. H1: *β* = 0.10, *z* = 3.56, *p* < 0.001; H3 vs. H1: *β* = 0.10, *z* = 2.16, *p* = 0.031) and increased over days (*β* = 0.14, *z* = 2.34, *p* = 0.019). The model also revealed a significant sex-day interaction (*β* = −0.15, *z* = −1.97, *p* = 0.049) which was not observed in Cohort 1. The zero-inflation part of the model also replicated the first cohort’s finding that SH significantly increased the probability of a complete lack of reward-seeking on the subsequent trial (*β* = 0.73, *z* = 3.08, *p* = 0.002; Supplementary Table 2), but did not replicate the significant effect of day (*p* = 0.759).

##### Avoidance behavior

The hurdle model again revealed no significant sex difference in the decision to leave the platform (*p* = 0.888; Supplementary Table 3). However, SH was a significant predictor (*β* = 0.66, *z* = 2.28, *p* = 0.023). The second part of the model (beta regression) confirmed the significant main effect of sex, (*β* = −0.60, *z* = −2.16, *p* = 0.031; Figure 3D; Supplementary Table 4). Avoidance was also predicted by SH (*β* = 0.47, *z* = 7.59, *p* < 0.001), day (*β* = −0.40, *z* = −4.03, *p* < 0.001), and trial number (*β* = −0.44, *z* = −5.14, *p* < 0.001), but the effect of weight did not replicate (*p* = 0.364). Additionally, handling group H2 showed significantly reduced avoidance (H2 vs. H1: *β* = −0.16, *z* = −2.90, *p* = 0.004). A significant sex-day interaction was also found (*β* = 0.38, *z* = 2.63, *p* = 0.008).

###### Latency to safety

The Gamma GLMM again revealed a significant main effect of sex, with males taking significantly longer to reach safety than females (*β* = 0.36, *z* = 2.17, *p* = 0.030; Figure 3E, Supplementary Table 5). Unlike the first cohort, weight was not a significant predictor in this model (*p* = 0.497). Consistent with Cohort 1, SH significantly reduced latency (*β* = −0.23, *z* = −6.31, *p* < 0.001), while later trials increased latency (*β* = 0.26, *z* = 5.03, *p* < 0.001). The model also identified a significant sex-day interaction (*β* = −0.32, *z* = −3.70, *p* < 0.001) and a main effect of day (*β* = 0.32, *z* = 5.34, *p* < 0.001).

###### Reward attentiveness

In contrast to the first cohort where a significant sex difference was found, the beta GLMM for reward attentiveness in the replication cohort did not show a significant main effect of sex (*p* = 0.502; Figure 3H; Supplementary Table 6). However, consistent with Cohort 1, attentiveness was significantly reduced by SH (*β* = −0.50, *z* = −9.33, *p* < 0.001). Additionally, both handling groups exhibited significantly higher attentiveness compared to the reference (H2 vs. H1: *β* = 0.19, *z* = 3.95, *p* < 0.001; H3 vs. H1: *β* = 0.19, *z* = 2.36, *p* = 0.018).

**Figure 4:**
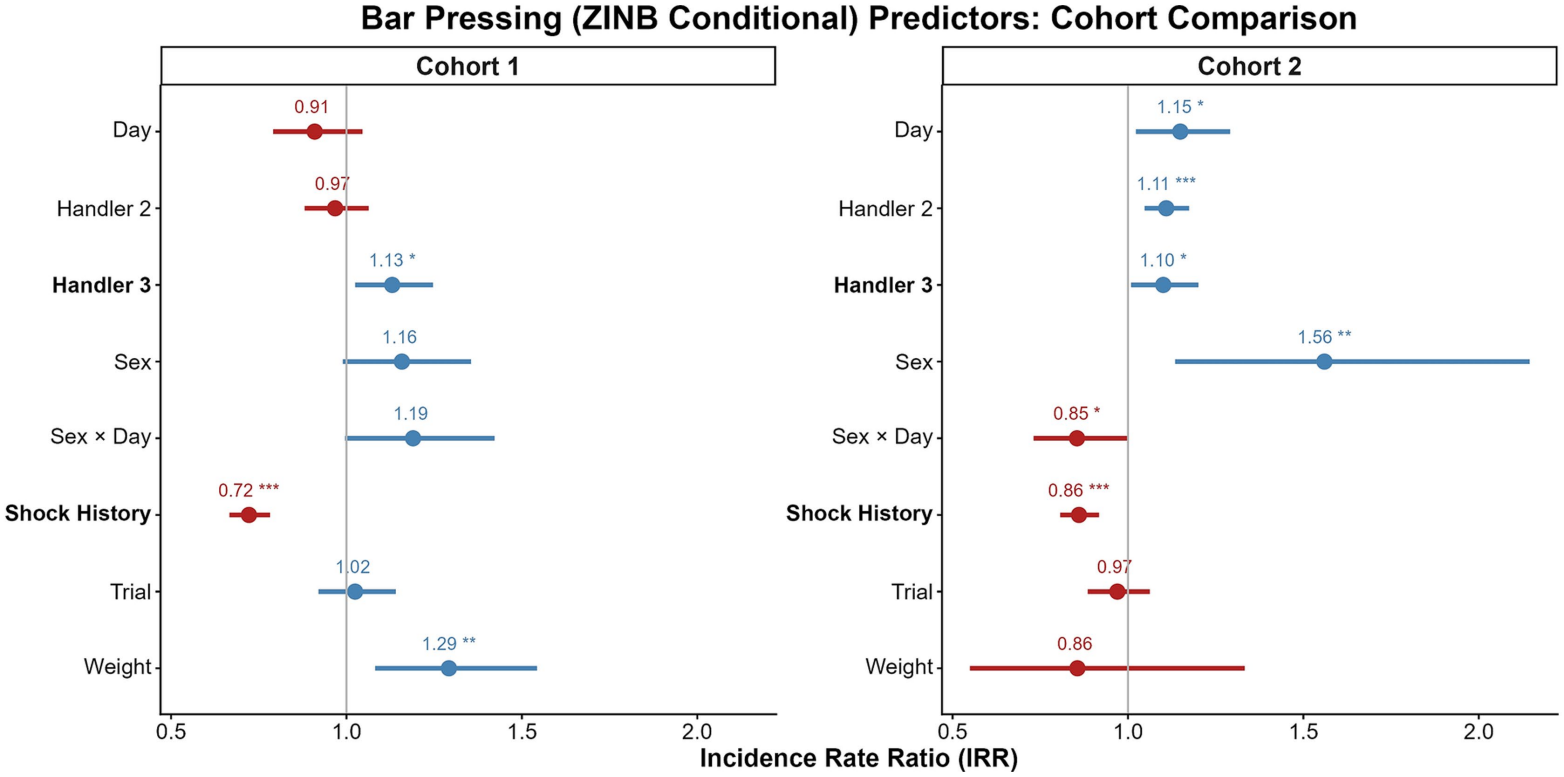
Comparison of predictors for bar pressing across cohorts. Forest plots display incidence rate ratios (IRRs) with 95% CI from the conditional count component of the zero-inflated negative binomial (ZINB) GLMMs for Cohort 1 (left) and Cohort 2 (right). IRRs represent the multiplicative effect of each predictor on the rate of bar pressing; values greater than 1 (blue) indicate a positive association, while values less than 1 (red) indicate a negative one. Predictors include: Sex (male vs. female reference), Day (session day), Tone (trial number), Handler2/3 (handler identity), shock history (prior shock that day), and Weight (*z*-scored body weight), as well as the Sex: Day interaction. Significant predictors with consistent directionality across cohorts are shown in bold. *p < 0.05, **p < 0.01, ***p < 0.001.

**Figure 5:**
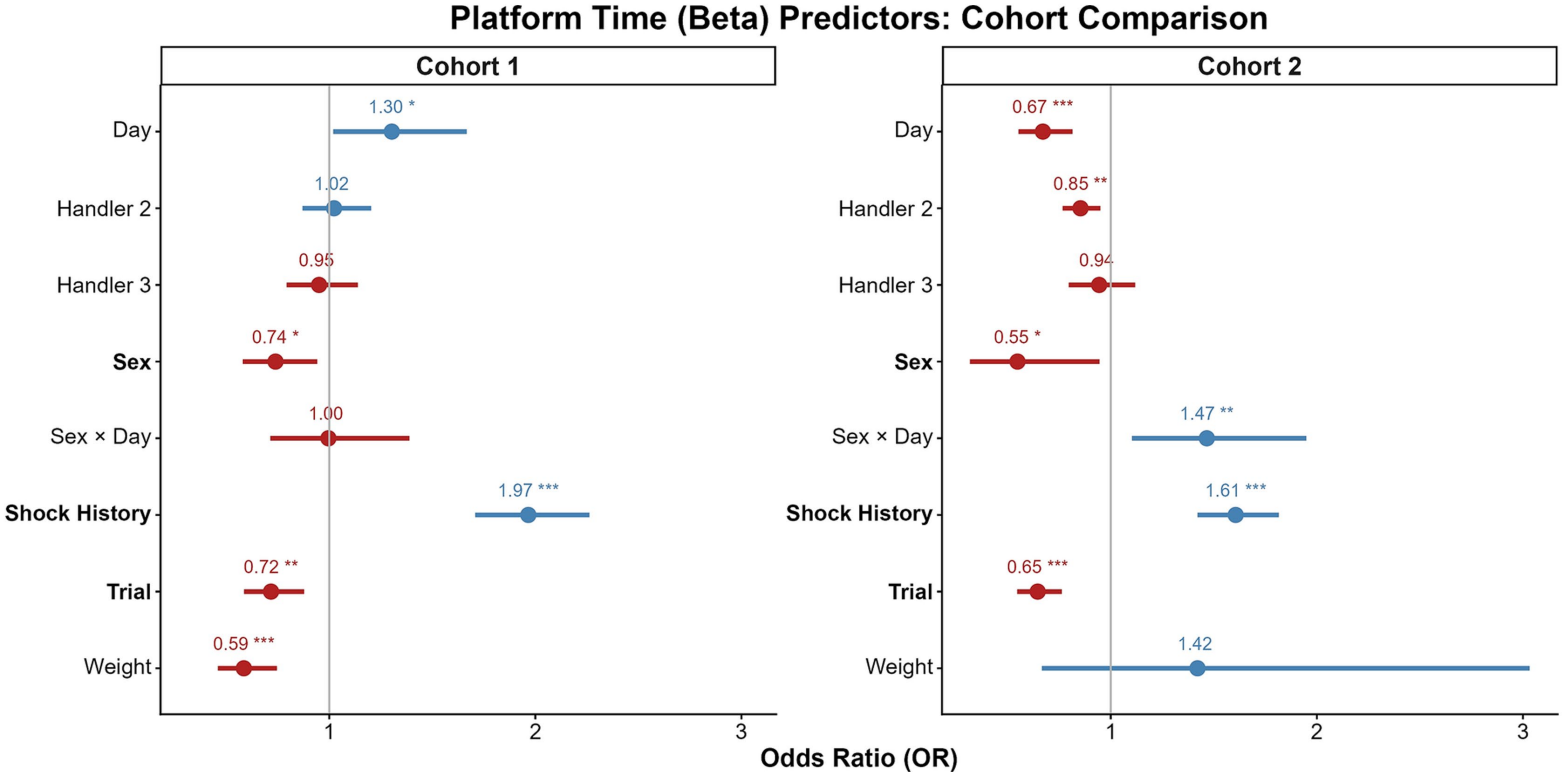
Comparison of predictors for avoidance behavior across cohorts. Forest plots display odds ratios (ORs) with 95% confidence intervals from the Beta component of the avoidance GLMMs for Cohort 1 (left) and Cohort 2 (right). ORs greater than 1 (blue) are associated with more time on the platform, while values less than 1 (red) are associated with less. The vertical line at 1 represents no effect. Significant predictors with consistent directionality across cohorts are shown in bold. *p < 0.05, **p < 0.01, ***p < 0.001.

**Figure 6:**
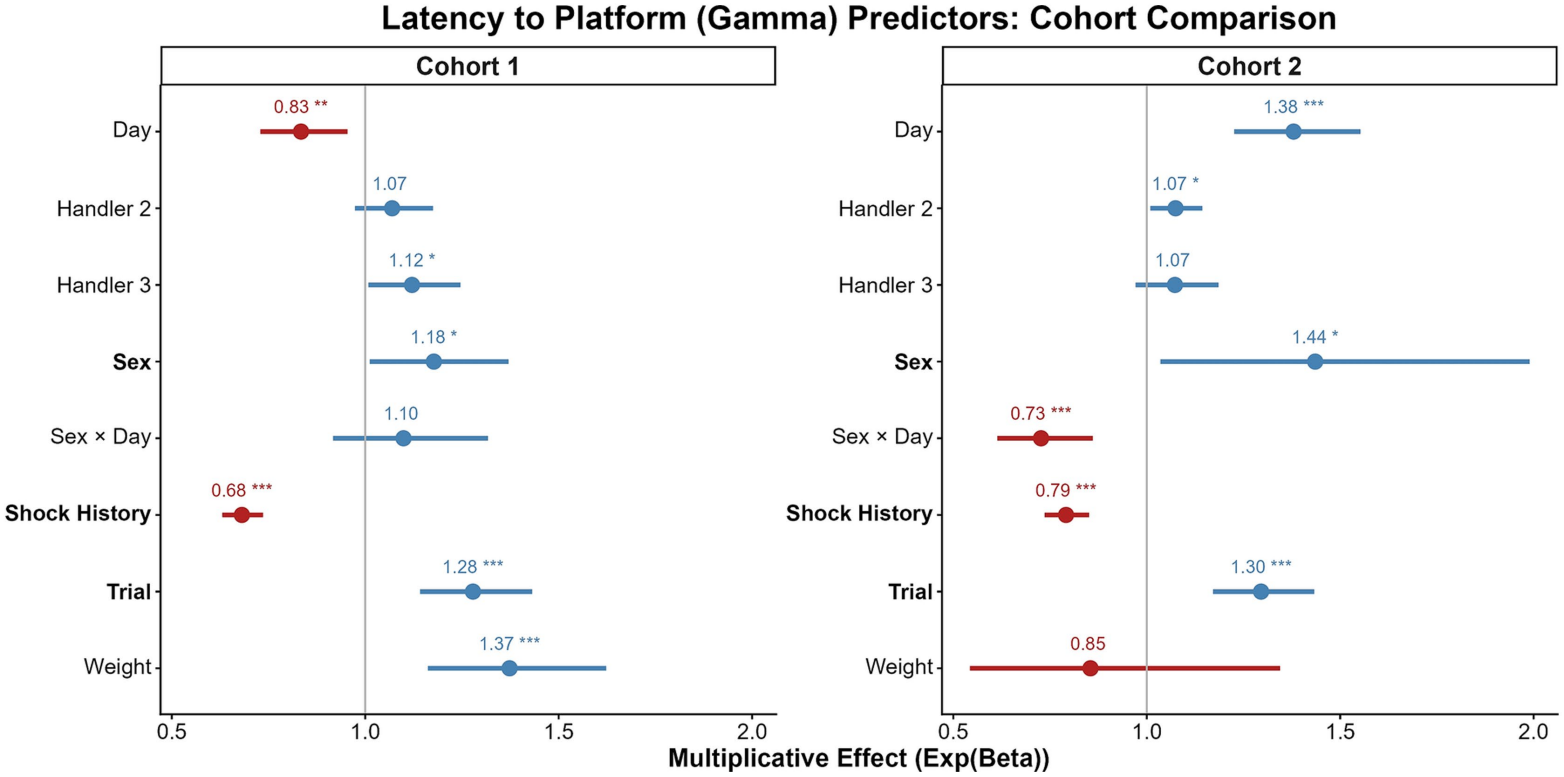
Comparison of predictors for latency to platform. Forest plot displaying exponentiated coefficients (multiplicative effects) and 95% confidence intervals from gamma GLMMs for cohort 1 (left) and cohort 2 (right). Values greater than 1 (blue) indicate increased latency to reach the safety platform; values less than 1 (red) indicate decreased latency. Predictors significant in both cohorts with consistent directionality are displayed in bold. Asterisks denote significance levels: *p < 0.05, **p < 0.01, ***p < 0.001.

**Figure 7:**
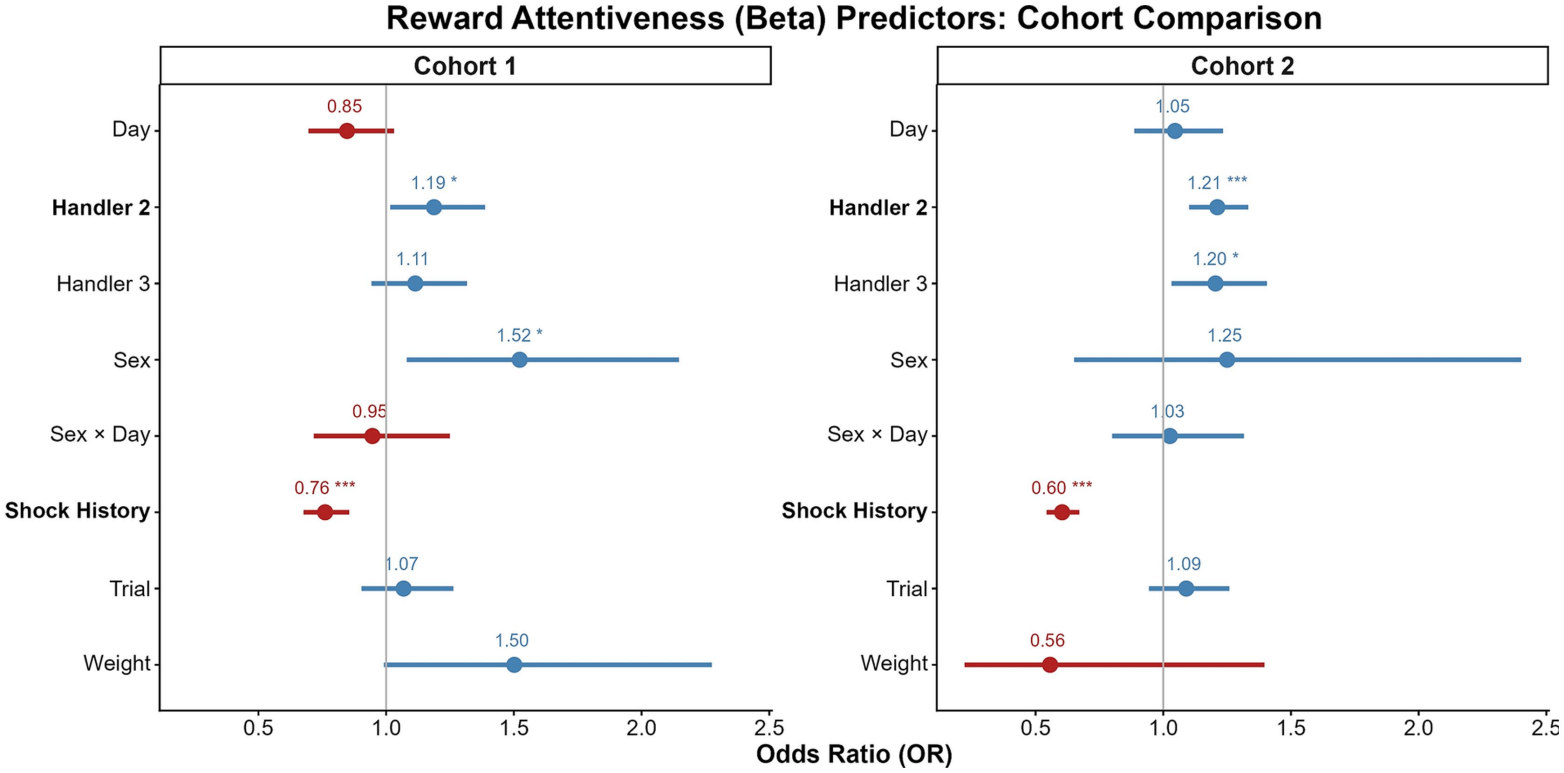
Comparison of predictors for reward attentiveness across cohorts. Forest plots display odds ratios (ORs) with 95% confidence intervals from the Beta GLMMs for cohort 1 (left) and cohort 2 (right). ORs greater than 1 (blue) indicate a positive association with attentiveness, while values less than 1 (red) indicate a negative association. The vertical line at 1 represents no effect. Significant predictors with consistent directionality across cohorts are shown in bold. *p < 0.05, **p < 0.01, ***p < 0.001.

The cross-cohort comparisons of our findings is summarized in Figures 4–7 and detailed in Supplementary Tables 1–6, which compare the model predictors for bar pressing, avoidance behavior, platform latency, and reward attentiveness between cohorts. The most replicable predictor was Shock History (having been shocked earlier in that session), but sex effects replicated, particularly for time on platform.

### Estrous cycle analysis

To explore whether avoidance and approach strategies were modulated by hormonal state, we conducted a supplementary analysis on the combined female dataset (n = 10) from the stable phase of behavior (days 20–25). Behavior was modeled as a function of a binary factor comparing the high-hormone phases (Proestrus/Estrus) against the low-hormone phases (Metestrus/Diestrus).

#### Reward-seeking behavior

**Figure 8:**
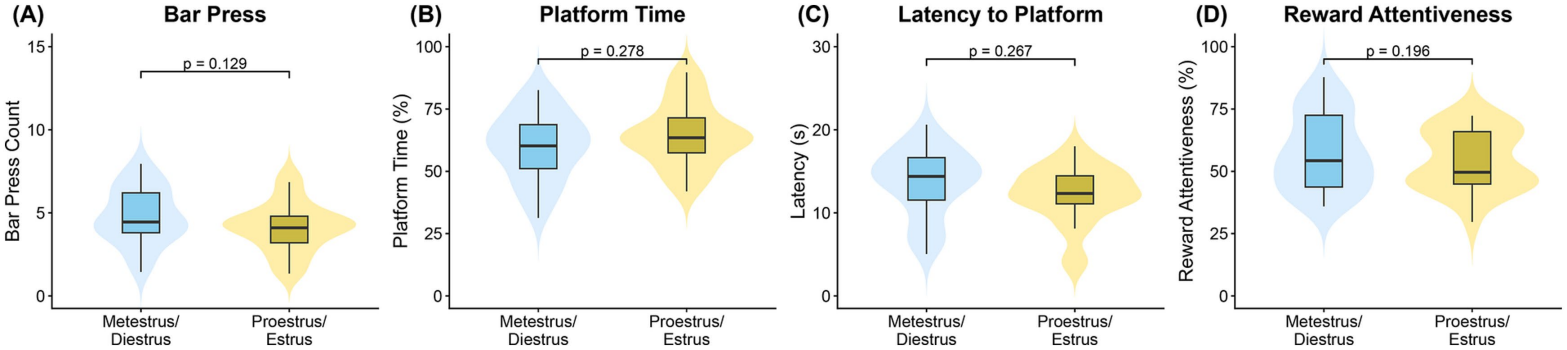
Estrous cycle does not impact stable-phase performance. Comparison of **(A)** bar press count, **(B)** platform time (%), **(C)** latency to platform (s), and **(D)** reward attentiveness (%) between low-hormone (metestrus/diestrus) and high-hormone (proestrus/estrus) states. Data are from the stable phase (days 20–25) for all females (*n* = 10, combined) and are visualized using violin plots with overlaid boxplots. *p*-values from the GLMM analysis are indicated.

In the ZINB model, estrous cycle phase (Proestrus/Estrus vs. Metestrus/Diestrus) was not a significant predictor of the number of bar presses (*p* = 0.129; Figure 8A; Supplementary Table 7). However, behavior remained sensitive to other variables; bar pressing was significantly reduced by SH (*β* = −0.25, *z* = −6.39, *p* < 0.001) and increased in handling group H3 (H3 vs. H1: *β* = 0.28, *z* = 6.01, *p* < 0.001). In the zero-inflation part of the model, cycle phase did not significantly influence the probability of engaging in reward-seeking (*p* = 0.981; Supplementary Table 8), but the probability of making zero presses was significantly increased by SH (*β* = 0.71, *z* = 3.46, *p* < 0.001), day (*β* = 0.69, *z* = 2.57, *p* = 0.010), and trial number (*β* = 0.83, *z* = 2.62, *p* = 0.009).

#### Avoidance behavior

The two-part hurdle-beta model for avoidance revealed no significant effect of estrous cycle phase. In the hurdle component, estrous phase did not influence the decision to remain on the platform (*p* = 0.991; Supplementary Table 9), which was instead positively associated with both day (*β* = 0.83, *z* = 2.82, *p* = 0.005) and trial number (*β* = 1.00, *z* = 2.90, *p* = 0.004). In the beta component, estrous phase did not influence the time spent avoiding (*p* = 0.278; Figure 8B; Supplementary Table 10). This duration was strongly increased by SH (*β* = 0.62, *z* = 9.02, *p* < 0.001) and decreased by trial number (*β* = −0.34, *z* = −3.58, *p* < 0.001).

#### Latency to safety

The Gamma GLMM for latency to platform revealed no significant effect of estrous cycle phase (*p* = 0.267; Figure 8C; Supplementary Table 11). Latency was, however, significantly modulated by other factors. Consistent with the primary analysis, SH significantly reduced latency to reach safety (*β* = −0.37, *z* = −8.85, *p* < 0.001). Latency increased significantly over days (*β* = 0.11, *z* = 2.37, *p* = 0.018) and within sessions as trial number progressed (*β* = 0.30, *z* = 5.35, *p* < 0.001). Additionally, handling group H3 showed significantly longer latencies compared to the reference (H3 vs. H1: *β* = 0.15, *z* = 2.86, *p* = 0.004).

#### Reward attentiveness

In the beta GLMM for reward attentiveness, no significant main effect of estrous cycle phase was found (*p* = 0.196; Figure 8D; Supplementary Table 12). However, attentiveness was significantly influenced by other factors; both handling groups H2 (H2 vs. H1: *β* = 0.24, *z* = 3.80, *p* < 0.001) and H3 (H3 vs. H1: *β* = 0.31, *z* = 3.80, *p* < 0.001) showed increased attentiveness compared to the reference group. As in other models, SH significantly reduced attentiveness (*β* = −0.34, *z* = −5.78, *p* < 0.001).

## Discussion

Confirming our initial hypothesis, the present study demonstrated robust and replicable sex differences in resolving approach-avoidance conflict, with females consistently prioritizing safety while males exhibit more persistent reward-seeking. These findings provide evidence for sex-specific behavioral strategies that align with and extend the existing literature on dimorphic threat responses and risk–reward preferences. This divergence seems consistent with other tasks where females often adopt stable, risk-averse strategies, while males are more influenced by recent outcomes and less deterred by negative consequences (Grissom and Reyes, 2019; Chen et al., 2021a; Grissom et al., 2024). Interpreting these results requires moving beyond a simple conclusion that females are “more responsive” to threats. Instead, as Shansky (2018) argues, these behavioral divergences likely represent qualitatively different, and potentially adaptive, strategies for navigating high-conflict situations.

A strength of the current study is the inclusion of a pre-registered replication cohort. While the significance of certain model predictors (e.g., body weight, handler) varied between cohorts - likely reflecting the sensitivity of behavioral tasks to environmental fluctuations - the core metrics defining the sexually dimorphic strategies demonstrated robust replicability. Specifically, the “safety-first” phenotype in females was confirmed across multiple dimensions: females spent significantly more time on the safety platform in both cohorts, males showed higher rates of lever-pressing under threat, and females evacuated the reward zone earlier in the threat window, as indicated by shorter latencies to reach safety. The convergence of these metrics across independent cohorts provides strong evidence that these sex differences reflect stable, biologically based differences in behavioral strategies.

Our results fit within a broader framework of active versus passive defensive behaviors. Preclinical literature consistently shows that when faced with a threat, males often adopt passive, immobile responses like freezing, whereas females tend to engage in active, mobile strategies (Dalla and Shors, 2009; Gruene et al., 2015a). This active strategy in females has been termed “anxioescapic behavior,” characterized by movement-based responses, such as darting or escape, rather than simple threat response potentiation (Shanazz et al., 2022; Bangasser and Cuarenta, 2021). The PMA task is exceptionally well-suited to capture this strategic divergence. By providing an unambiguous safe location, it allows for the expression of an active avoidance response that might be masked in paradigms that rely solely on freezing. The greater time females spent on the platform, therefore, likely reflects the expression of this coping strategy rather than a simple inability to approach the reward.

The high-conflict nature of our paradigm, where rewards were only available under imminent threat, is crucial for revealing these sex-specific priorities. Rather than simply reflecting greater anxiety, this strategic divide may represent a sex difference in decision-making, as females in both human and animal studies show a strong tendency to avoid options associated with frequent negative outcomes (van den Bos et al., 2013; Grissom and Reyes, 2019). This strategic divide is not unique to PMA and has been observed in other approach-avoidance models. For instance, in the Vogel conflict test, where water-deprived rats are punished with a shock for drinking, females also display greater behavioral inhibition, accepting significantly fewer punished responses than males (Basso et al., 2011). This parallel finding reinforces the idea that females, across different types of conflict, adopt a more cautious, safety-prioritized strategy. Notably, Basso et al. (2011) also reported a pharmacological dissociation: while classic benzodiazepines were anxiolytic in both sexes, other compounds like SSRIs and buspirone only produced anxiolytic-like effects in males. This pharmacological dissociation provides an important indication: while some core circuits for processing conflict may be shared between sexes, the systems that modulate these circuits and ultimately shape the behavioral strategy seem sexually dimorphic. This suggests that the decision-making process involves both shared and sex-specific aspects of neurocircuitry and circuit functioning, which may be differentially influenced by neuromodulatory systems. It is important to note that the “safety-first” strategy could partially reflect sex differences in pain sensitivity, as females generally exhibit lower thresholds than males (Mogil and Bailey, 2010; Sorge and Totsch, 2017).

While our avoidance/approach metrics differed between sexes, reward attentiveness showed no consistent sex difference across cohorts. This suggests that the strategic divergence may not lie in the amount of deliberation itself; both sexes appear to engage in a similar degree of conflict processing, weighing the value of the reward against the imminent threat. The difference, therefore, appears to be in the outcome of this deliberative process. Despite their engagement with the reward, females seem to more consistently suppress the final motor-approach response in favor of a safety-prioritized strategy. This dissociation between deliberation and action strongly supports the hypothesis that males and females adopt qualitatively different strategies for resolving the conflict (Shansky, 2018). Thus, the dimorphism observed here may not arise from the assessment of the conflict per se, but rather in the downstream decision-making circuits that translate the output of that assessment into a behavioral choice.

These distinct behavioral strategies likely arise from sexually dimorphic neural and endocrine mechanisms. The work of Bravo-Rivera et al. (2021), which characterized these behavioral phenotypes in rats, offers a potential neural basis for our findings. They demonstrated that an avoidance-preferring strategy, which we observed in females, was associated with heightened activity in the amygdala, a key hub for threat response. Other threat-related behaviors, such as conditioned freezing and escape, have been linked to amygdala and particularly its reciprocal interactions with prefrontal cortex (Diehl et al., 2024; Plas et al., 2024). Those circuits may also be dimorphic – one study suggested that females and males “solve” extinction learning differently, with females depending less on infralimbic cortex (Gruene et al., 2015b). Thus, identifying the neural basis of sexually dimorphic avoidance likely requires simultaneous measurement of multiple structures within the canonical fear circuitry.

Furthermore, gonadal hormones may profoundly shape these circuits. This shaping occurs not only through the immediate influence of circulating hormones in adulthood (activational effects) but also through the permanent organizing of neural pathways during critical developmental periods (organizational effects) (McCarthy et al., 2012, 2015). The influence of high estrogen levels, for instance, on facilitating the extinction of conditioned fear would be an activational effect (Graham and Milad, 2014; Ramikie and Ressler, 2018). However, findings from Halcomb et al. (2024) in a modified PMA task revealed that persistent avoidance in female mice was significantly reduced by a glucocorticoid receptor (GR) antagonist administered during learning. This suggests that GR activation during the acquisition of the threat association, rather than activational effects of circulating gonadal hormones alone, may be a key mechanism that defines and maintains this avoidance strategy in females.

While our study did not find a significant modulation by the estrous cycle in our exploratory analysis, the role of hormonal fluctuations remains an important consideration (Li and Graham, 2017). This is consistent with findings from both the modified PMA task (Halcomb et al., 2024) and the Vogel test (Basso et al., 2011), where the estrous cycle did not influence avoidance behavior. The influence of the estrous cycle can be, however, task-dependent, as Carvalho et al. (2021) found it impacted fear-potentiated startle but not conditioned freezing. Together, this suggests that while ovarian hormones are critical for the developmental organization of these sexually dimorphic circuits, the expression of a stable, learned behavioral strategy in a high-conflict task may be less sensitive to acute fluctuations than other measures of fear-like responses. However, it is important to note that our findings characterize the safety-first strategy specifically in nulliparous females. Literature on maternal behavior suggests that lactation and reproductive experience can induce a risk-prone phenotype, potentially to ensure resource acquisition for offspring (Ferreira et al., 1989; Faraji et al., 2024), suggesting that the safety-prioritization observed here is likely a state-dependent adaptation rather than an inherent sex difference.

A limitation of the current study is that the neurobiological mechanisms underlying these sex-specific strategies were not directly assessed. Future studies incorporating neural recordings and causal manipulations - particularly targeting oscillatory dynamics within amygdala-prefrontal circuitry, which has been implicated in both conflict processing and sexually dimorphic threat responses (Gruene et al., 2015b; Bravo-Rivera et al., 2021; Totty and Maren, 2022) - will be necessary to validate the proposed mechanisms and enhance translational relevance. A consideration inherent to conflict paradigms is that multiple decision-making components are integrated within the task, and the current design does not permit complete dissociation of all underlying processes. Nevertheless, our data suggest that the sex difference emerges specifically under conflict: males and females showed equivalent reward-seeking during the non-conflict Light Cue phase, similar reward attentiveness, and the statistical modeling revealed that sex predicts for how long animals will endure rather than whether they approach. Future studies utilizing shock titration to equate subjective cost across subjects, progressive ratio schedules, or sucrose preference assays could further isolate the specific contributions of punishment sensitivity, reward valuation, and risk tolerance.

In conclusion, we show that male and female rats employ fundamentally different strategies to resolve approach-avoidance conflict. Females adopt a safety-first strategy, consistent with a more active coping style that seems more resistant to change. Males, in contrast, employ a risk-prone, reward-oriented strategy. The translational importance of these findings is significant. The persistent avoidance shown by females mirrors a core clinical feature of anxiety and trauma-related disorders that is often a primary target for intervention. Understanding the distinct neurobiological pathways and mechanisms that support these strategies is a critical step toward developing more effective, personalized treatments. While acknowledging challenges in translating circuit-level findings across species (de Oliveira et al., 2021) the next critical step is to characterize how this circuit activity differs between sexes. This characterization may provide the necessary biomarkers for developing targeted neuromodulation therapies, such as deep brain stimulation, to ameliorate persistent avoidance.

## Data Availability

The raw data supporting the conclusions of this article will be made available by the authors, without undue reservation.
