# Supplementary material for: Female rats adopt a safety-first strategy in a high-conflict platform mediated avoidance task
Source: Front Behav Neurosci. 2026 Feb 18;20:1758605. doi: 10.3389/fnbeh.2026.1758605 (PMC12957229; doi:10.3389/fnbeh.2026.1758605)
Supplement: Supplementary file 1 [file Table_1.docx]

Supplementary Material

**Females Rats Adopt a Safety-First Strategy in a High-Conflict Platform Mediated Avoidance Task**

**Adriano E. Reimer*, Christina J. Li*, Steven M. Hu*, Delilah Pineda*, Jason L. Chang, Michael R. Angstman, Evan M. Dastin-van Rijn, Alik S. Widge**

***Correspondence: Alik S. Widge: awidge@umn.edu**

**Supplementary Table 1.** Reward-Seeking Frequency: ZINB Model – Conditional Count Component. Comparisons between Cohort 1 and Cohort 2 of predictors for the number of bar presses (during trials where engagement occurred). Estimates are derived from the conditional negative binomial component of the Zero-Inflated Negative Binomial (ZINB) model. The model includes fixed effects for Sex, Day, Trial, Handlers, Shock History, Weight, and the Sex × Day interaction.

| **Bar Presses - All Predictors Cohort Comparison ZINB GLMM (Conditional Negative Binomial)** | | | | | | | | | | |
| --- | --- | --- | --- | --- | --- | --- | --- | --- | --- | --- |
|  | **Cohort 1** | | | | | **Cohort 2** | | | | |
| **Parameter** | **β** | **exp(β)** | **SE** | **z** | **P** | **β** | **exp(β)** | **SE** | **z** | **P** |
| Intercept | 1.70 | 5.48 | 0.09 | 18.93 | <0.001 | 1.62 | 5.08 | 0.15 | 10.92 | <0.001 |
| Day | -0.09 | 0.91 | 0.07 | -1.33 | 0.182 | 0.14 | 1.15 | 0.06 | 2.34 | 0.019 |
| Handler 2 | -0.03 | 0.97 | 0.05 | -0.69 | 0.493 | 0.10 | 1.11 | 0.03 | 3.56 | <0.001 |
| **Handler 3** | **0.12** | **1.13** | **0.05** | **2.45** | **0.014** | **0.10** | **1.10** | **0.04** | **2.16** | **0.031** |
| Sex (M x F) | 0.15 | 1.16 | 0.08 | 1.83 | 0.067 | 0.44 | 1.56 | 0.16 | 2.74 | 0.006 |
| Sex × Day | 0.17 | 1.19 | 0.09 | 1.91 | 0.056 | -0.16 | 0.85 | 0.08 | -1.97 | 0.049 |
| **Shock History** | **-0.33** | **0.72** | **0.04** | **-7.97** | **<0.001** | **-0.15** | **0.86** | **0.03** | **-4.59** | **<0.001** |
| Trial | 0.02 | 1.02 | 0.05 | 0.44 | 0.658 | -0.03 | 0.97 | 0.05 | -0.66 | 0.508 |
| Weight | 0.26 | 1.29 | 0.09 | 2.83 | 0.005 | -0.16 | 0.86 | 0.23 | -0.69 | 0.491 |
|  | | | | | | | | | | |

**Supplementary Table 2.** Probability of Non-Engagement: ZINB Model – Zero-Inflation Component. Comparisons between Cohort 1 and Cohort 2 of predictors for the probability of performing zero bar presses (complete non-engagement). Estimates are derived from the logistic zero-inflation component of the ZINB model. The model includes fixed effects for Sex, Day, Trial, Handlers, Shock History, Weight, and the Sex × Day interaction.

| **Bar Presses - All Predictors Cohort Comparison - ZINB GLMM (Zero-Inflation)** | | | | | | | | | | |
| --- | --- | --- | --- | --- | --- | --- | --- | --- | --- | --- |
|  | **Cohort 1** | | | | | **Cohort 2** | | | | |
| **Parameter** | **β** | **exp(β)** | **SE** | **z** | **P** | **β** | **exp(β)** | **SE** | **z** | **P** |
| Intercept | -1.66 | 0.19 | 0.61 | -2.71 | 0.007 | -2.89 | 0.06 | 0.75 | -3.85 | <0.001 |
| Day | 0.93 | 2.55 | 0.36 | 2.60 | 0.009 | 0.12 | 1.13 | 0.39 | 0.31 | 0.759 |
| Handler 2 | -0.09 | 0.91 | 0.33 | -0.28 | 0.780 | 0.38 | 1.47 | 0.27 | 1.42 | 0.156 |
| Handler 3 | -0.08 | 0.93 | 0.34 | -0.22 | 0.822 | 0.40 | 1.49 | 0.38 | 1.06 | 0.289 |
| Sex (M x F) | -0.96 | 0.38 | 0.61 | -1.58 | 0.115 | 0.10 | 1.11 | 0.80 | 0.13 | 0.899 |
| Sex × Day | -0.38 | 0.68 | 0.60 | -0.63 | 0.527 | -1.43 | 0.24 | 0.69 | -2.07 | 0.038 |
| **Shock History** | **0.66** | **1.93** | **0.23** | **2.84** | **0.005** | **0.73** | **2.08** | **0.24** | **3.08** | **0.002** |
| Trial | 0.43 | 1.54 | 0.34 | 1.27 | 0.204 | -0.05 | 0.95 | 0.38 | -0.14 | 0.892 |
| Weight | -0.87 | 0.42 | 0.67 | -1.30 | 0.193 | -0.09 | 0.91 | 1.06 | -0.09 | 0.931 |

**Supplementary Table 3.** Probability of Leaving the Platform: Hurdle-Beta Model – Binomial Component. Comparisons between Cohort 1 and Cohort 2 of predictors for the probability of leaving the safe platform at any point during the tone (versus remaining on it for the full duration). Estimates are derived from the binomial hurdle component of the Hurdle-Beta model. The model includes fixed effects for Sex, Day, Trial, Handlers, Shock History, Weight, and the Sex × Day interaction.

| **Platform Time All Predictors Cohort Comparison Hurdle-Beta GLMM (Hurdle Binomial)** | | | | | | | | | | |
| --- | --- | --- | --- | --- | --- | --- | --- | --- | --- | --- |
|  | **Cohort 1** | | | | | **Cohort 2** | | | | |
| **Parameter** | **β** | **exp(β)** | **SE** | **z** | **P** | **β** | **exp(β)** | **SE** | **z** | **P** |
| Intercept | -1.49 | 0.23 | 0.58 | -2.59 | 0.010 | -4.10 | 0.02 | 0.65 | -6.30 | <0.001 |
| Day | 0.59 | 1.80 | 0.38 | 1.55 | 0.122 | 0.76 | 2.13 | 0.45 | 1.69 | 0.092 |
| Handler 2 | -0.49 | 0.61 | 0.36 | -1.34 | 0.180 | 0.47 | 1.61 | 0.33 | 1.45 | 0.146 |
| Handler 3 | -0.38 | 0.69 | 0.36 | -1.03 | 0.301 | -1.12 | 0.33 | 0.76 | -1.47 | 0.142 |
| Sex (M x F) | -0.90 | 0.41 | 0.59 | -1.54 | 0.124 | 0.09 | 1.10 | 0.68 | 0.14 | 0.888 |
| Sex × Day | -0.78 | 0.46 | 0.70 | -1.12 | 0.265 | -1.48 | 0.23 | 0.84 | -1.76 | 0.078 |
| Shock History | 0.01 | 1.01 | 0.27 | 0.02 | 0.983 | 0.66 | 1.94 | 0.29 | 2.28 | 0.023 |
| Trial | 0.46 | 1.58 | 0.38 | 1.22 | 0.222 | 0.37 | 1.44 | 0.45 | 0.82 | 0.415 |
| Weight | -0.96 | 0.38 | 0.60 | -1.60 | 0.111 | 0.54 | 1.71 | 0.77 | 0.70 | 0.485 |
|  | | | | | | | | | | |

**Supplementary Table 4.** Proportion of Time on Platform: Hurdle-Beta Model – Beta Regression Component. Comparisons between Cohort 1 and Cohort 2 of predictors for the proportion of trial time spent on the platform. Estimates are derived from the beta regression component of the Hurdle-Beta model. The model includes fixed effects for Sex, Day, Trial, Handlers, Shock History, Weight, and the Sex × Day interaction.

| **Platform Time - All Predictors Cohort Comparison - Hurdle-Beta GLMM (Conditional Beta)** | | | | | | | | | | |
| --- | --- | --- | --- | --- | --- | --- | --- | --- | --- | --- |
|  | **Cohort 1** | | | | | **Cohort 2** | | | | |
| **Parameter** | **β** | **exp(β)** | **SE** | **z** | **P** | **β** | **exp(β)** | **SE** | **z** | **P** |
| Intercept | 0.45 | 1.57 | 0.13 | 3.34 | <0.001 | 0.50 | 1.65 | 0.26 | 1.95 | 0.051 |
| Day | 0.27 | 1.30 | 0.13 | 2.11 | 0.035 | -0.40 | 0.67 | 0.10 | -4.03 | <0.001 |
| Handler 2 | 0.02 | 1.02 | 0.08 | 0.28 | 0.781 | -0.16 | 0.85 | 0.05 | -2.90 | 0.004 |
| Handler 3 | -0.05 | 0.95 | 0.09 | -0.55 | 0.583 | -0.06 | 0.94 | 0.09 | -0.67 | 0.505 |
| **Sex (M x F)** | **-0.30** | **0.74** | **0.12** | **-2.44** | **0.015** | **-0.60** | **0.55** | **0.28** | **-2.16** | **0.031** |
| Sex × Day | 0.00 | 1.00 | 0.17 | -0.02 | 0.981 | 0.38 | 1.47 | 0.15 | 2.63 | 0.008 |
| **Shock History** | **0.68** | **1.97** | **0.07** | **9.43** | **<0.001** | **0.47** | **1.61** | **0.06** | **7.59** | **<0.001** |
| **Trial** | **-0.33** | **0.72** | **0.10** | **-3.22** | **0.001** | **-0.44** | **0.65** | **0.09** | **-5.14** | **<0.001** |
| Weight | -0.54 | 0.59 | 0.12 | -4.33 | <0.001 | 0.35 | 1.42 | 0.39 | 0.91 | 0.364 |

**Supplementary Table 5.** Latency to Safety: Gamma Regression Model. Comparisons between Cohort 1 and Cohort 2 of predictors for the latency to enter the safe platform zone. Estimates are derived from the Gamma GLMM with a log link function. The model includes fixed effects for Sex, Day, Trial, Handlers, Shock History, Weight, and the Sex × Day interaction.

| **Latency to Platform - All Predictors Cohort Comparison - Gamma GLMM** | | | | | | | | | | |
| --- | --- | --- | --- | --- | --- | --- | --- | --- | --- | --- |
|  | **Cohort 1** | | | | | **Cohort 2** | | | | |
| **Parameter** | **β** | **exp(β)** | **SE** | **z** | **P** | **β** | **exp(β)** | **SE** | **z** | **P** |
| Intercept | 2.37 | 10.68 | 0.09 | 27.67 | <0.001 | 2.35 | 10.45 | 0.15 | 15.40 | <0.001 |
| Day | -0.18 | 0.83 | 0.07 | -2.64 | 0.008 | 0.32 | 1.38 | 0.06 | 5.34 | <0.001 |
| Handler 2 | 0.07 | 1.07 | 0.05 | 1.40 | 0.162 | 0.07 | 1.07 | 0.03 | 2.26 | 0.024 |
| Handler 3 | 0.11 | 1.12 | 0.05 | 2.11 | 0.035 | 0.07 | 1.07 | 0.05 | 1.38 | 0.166 |
| **Sex (M x F)** | **0.16** | **1.18** | **0.08** | **2.11** | **0.035** | **0.36** | **1.44** | **0.17** | **2.17** | **0.030** |
| Sex × Day | 0.09 | 1.10 | 0.09 | 1.02 | 0.308 | -0.32 | 0.73 | 0.09 | -3.70 | <0.001 |
| **Shock History** | **-0.38** | **0.68** | **0.04** | **-9.74** | **<0.001** | **-0.23** | **0.79** | **0.04** | **-6.31** | **<0.001** |
| **Trial** | **0.25** | **1.28** | **0.06** | **4.26** | **<0.001** | **0.26** | **1.30** | **0.05** | **5.03** | **<0.001** |
| Weight | 0.32 | 1.37 | 0.09 | 3.72 | <0.001 | -0.16 | 0.85 | 0.23 | -0.68 | 0.497 |
|  | | | | | | | | | | |

**Supplementary Table 6.** Reward Attentiveness: Beta Regression Model. Comparisons between Cohort 1 and Cohort 2 of predictors for the proportion of total trial time the animal spent oriented toward the reward zone. Estimates are derived from the Beta GLMM. The model includes fixed effects for Sex, Day, Trial, Handlers, Shock History, Weight, and the Sex × Day interaction.

| **Reward Attentiveness - All Predictors Cohort Comparison - Beta GLMM** | | | | | | | | | | |
| --- | --- | --- | --- | --- | --- | --- | --- | --- | --- | --- |
|  | **Cohort 1** | | | | | **Cohort 2** | | | | |
| **Parameter** | **β** | **exp(β)** | **SE** | **z** | **P** | **β** | **exp(β)** | **SE** | **z** | **P** |
| Intercept | -0.10 | 0.91 | 0.19 | -0.52 | 0.604 | 0.65 | 1.92 | 0.30 | 2.15 | 0.032 |
| Day | -0.17 | 0.85 | 0.10 | -1.66 | 0.098 | 0.05 | 1.05 | 0.08 | 0.53 | 0.593 |
| **Handler 2** | **0.17** | **1.19** | **0.08** | **2.16** | **0.031** | **0.19** | **1.21** | **0.05** | **3.95** | **<0.001** |
| Handler 3 | 0.11 | 1.11 | 0.09 | 1.27 | 0.205 | 0.19 | 1.20 | 0.08 | 2.36 | 0.018 |
| Sex (M x F) | 0.42 | 1.52 | 0.18 | 2.40 | 0.016 | 0.22 | 1.25 | 0.33 | 0.67 | 0.502 |
| Sex × Day | -0.06 | 0.95 | 0.14 | -0.39 | 0.697 | 0.03 | 1.03 | 0.13 | 0.20 | 0.840 |
| **Shock History** | **-0.27** | **0.76** | **0.06** | **-4.56** | **<0.001** | **-0.50** | **0.60** | **0.05** | **-9.33** | **<0.001** |
| Trial | 0.07 | 1.07 | 0.09 | 0.77 | 0.439 | 0.09 | 1.09 | 0.07 | 1.17 | 0.240 |
| Weight | 0.41 | 1.50 | 0.21 | 1.92 | 0.055 | -0.59 | 0.56 | 0.47 | -1.25 | 0.212 |
|  | | | | | | | | | | |

**Supplementary Table 7.** Estrous Cycle – Reward-Seeking Frequency: ZINB Model – Conditional Count Component. Predictors for the number of bar presses (during trials where engagement occurred) in the combined female cohort. Estimates are derived from the conditional negative binomial component of the Zero-Inflated Negative Binomial (ZINB) model. The model includes fixed effects for Estrous Phase (High vs Low), Day, Trial, Handlers, and Shock History.

| **Parameter** | **β** | **exp(β)** | **SE** | **z** | **P** |
| --- | --- | --- | --- | --- | --- |
| Intercept | 1.72 | 5.56 | 0.10 | 17.44 | <0.001 |
| Day | 0.06 | 1.06 | 0.04 | 1.34 | 0.180 |
| Estrous Phase (High vs Low) | -0.05 | 0.95 | 0.03 | -1.52 | 0.129 |
| Handler 2 | 0.07 | 1.08 | 0.04 | 1.91 | 0.056 |
| Handler 3 | 0.28 | 1.33 | 0.05 | 6.01 | <0.001 |
| Shock History | -0.25 | 0.78 | 0.04 | -6.39 | <0.001 |
| Trial | -0.10 | 0.91 | 0.05 | -1.89 | 0.058 |

**Supplementary Table 8.** Estrous Cycle – Probability of Non-Engagement: ZINB Model – Zero-Inflation Component. Predictors for the probability of performing zero bar presses (complete non-engagement) in the combined female cohort. Estimates are derived from the logistic zero-inflation component of the ZINB model. The model includes fixed effects for Estrous Phase (High vs Low), Day, Trial, Handlers, Shock History, and Weight.

| **Parameter** | **β** | **exp(β)** | **SE** | **z** | **P** |
| --- | --- | --- | --- | --- | --- |
| Intercept | -3.22 | 0.04 | 0.91 | -3.55 | <0.001 |
| Day | 0.69 | 1.99 | 0.27 | 2.57 | 0.010 |
| Estrous Phase (High vs Low) | -0.01 | 0.99 | 0.23 | -0.02 | 0.981 |
| Handler 2 | 0.21 | 1.23 | 0.27 | 0.75 | 0.451 |
| Handler 3 | 0.32 | 1.38 | 0.30 | 1.08 | 0.281 |
| Shock History | 0.71 | 2.03 | 0.20 | 3.46 | <0.001 |
| Trial | 0.83 | 2.29 | 0.32 | 2.62 | 0.009 |
| Weight | 0.08 | 1.08 | 1.41 | 0.06 | 0.956 |

**Supplementary Table 9**. Estrous Cycle – Probability of Leaving the Platform: Hurdle-Beta Model – Binomial Component. Predictors for the probability of leaving the safe platform at any point during a trial (versus remaining on it for the full duration) in the combined female cohort. Estimates are derived from the binomial hurdle component of the Hurdle-Beta model. The model includes fixed effects for Estrous Phase (High vs Low), Day, Trial, Handlers, Shock History, and Weight.

| **Parameter** | **β** | **exp(β)** | **SE** | **z** | **P** |
| --- | --- | --- | --- | --- | --- |
| Intercept | -3.59 | 0.03 | 0.90 | -4.00 | <0.001 |
| Day | 0.83 | 2.29 | 0.29 | 2.82 | 0.005 |
| Estrous Phase (High vs Low) | 0.00 | 1.00 | 0.25 | 0.01 | 0.991 |
| Handler 2 | 0.13 | 1.14 | 0.30 | 0.43 | 0.664 |
| Handler 3 | -0.45 | 0.64 | 0.38 | -1.20 | 0.230 |
| Shock History | 0.31 | 1.36 | 0.23 | 1.35 | 0.179 |
| Trial | 1.00 | 2.72 | 0.34 | 2.90 | 0.004 |
| Weight | 0.05 | 1.05 | 1.35 | 0.04 | 0.970 |

**Supplementary Table 10.** Estrous Cycle – Proportion of Time on Platform: Hurdle-Beta Model – Beta Regression Component. Predictors for the proportion of trial time spent on the platform (during trials where the animal left the platform at least once) in the combined female cohort. Estimates are derived from the beta regression component of the Hurdle-Beta model. The model includes fixed effects for Estrous Phase (High vs Low), Day, Trial, Handlers, Shock History, and Weight.

| **Parameter** | **β** | **exp(β)** | **SE** | **z** | **P** |
| --- | --- | --- | --- | --- | --- |
| Intercept | 0.21 | 1.23 | 0.28 | 0.75 | 0.454 |
| Day | -0.12 | 0.88 | 0.08 | -1.57 | 0.116 |
| Estrous Phase (High vs Low) | 0.06 | 1.07 | 0.06 | 1.08 | 0.278 |
| Handler 2 | -0.08 | 0.93 | 0.07 | -1.08 | 0.281 |
| Handler 3 | -0.17 | 0.84 | 0.09 | -1.90 | 0.058 |
| Shock History | 0.62 | 1.86 | 0.07 | 9.02 | <0.001 |
| Trial | -0.34 | 0.72 | 0.09 | -3.58 | <0.001 |
| Weight | 0.29 | 1.33 | 0.45 | 0.64 | 0.520 |

**Supplementary Table 11.** Estrous Cycle – Latency to Safety: Gamma Regression Model. Predictors for the latency to enter the safe platform zone in the combined female cohort. Estimates are derived from the Gamma GLMM with a log link function. The model includes fixed effects for Estrous Phase (High vs Low), Day, Trial, Handlers, Shock History, and Weight.

| **Parameter** | **β** | **exp(β)** | **SE** | **z** | **P** |
| --- | --- | --- | --- | --- | --- |
| Intercept | 2.43 | 11.36 | 0.19 | 13.09 | <0.001 |
| Day | 0.11 | 1.12 | 0.05 | 2.37 | 0.018 |
| Estrous Phase (High vs Low) | 0.04 | 1.04 | 0.04 | 1.11 | 0.267 |
| Handler 2 | 0.06 | 1.06 | 0.04 | 1.55 | 0.121 |
| Handler 3 | 0.15 | 1.17 | 0.05 | 2.86 | 0.004 |
| Shock History | -0.37 | 0.69 | 0.04 | -8.85 | <0.001 |
| Trial | 0.30 | 1.35 | 0.06 | 5.35 | <0.001 |
| Weight | -0.09 | 0.92 | 0.30 | -0.29 | 0.772 |

**Supplementary Table 12.** Estrous Cycle – Reward Attentiveness: Beta Regression Model. Predictors for the proportion of total trial time the animal spent oriented toward the reward zone in the combined female cohort. Estimates are derived from the Beta GLMM. The model includes fixed effects for Estrous Phase (High vs Low), Day, Trial, Handlers, Shock History, and Weight.

| **Parameter** | **β** | **exp(β)** | **SE** | **z** | **P** |
| --- | --- | --- | --- | --- | --- |
| Intercept | 0.49 | 1.64 | 0.34 | 1.47 | 0.140 |
| Day | -0.05 | 0.96 | 0.07 | -0.67 | 0.504 |
| Estrous Phase (High vs Low) | -0.07 | 0.93 | 0.05 | -1.29 | 0.196 |
| Handler 2 | 0.24 | 1.27 | 0.06 | 3.79 | <0.001 |
| Handler 3 | 0.31 | 1.37 | 0.08 | 3.80 | <0.001 |
| Shock History | -0.34 | 0.71 | 0.06 | -5.78 | <0.001 |
| Trial | -0.01 | 0.99 | 0.08 | -0.18 | 0.854 |
| Weight | -0.39 | 0.68 | 0.55 | -0.71 | 0.480 |
